# Hematopoietic cell transplantation for asymptomatic X-linked lymphoproliferative syndrome type 1

**DOI:** 10.1186/s13223-018-0306-1

**Published:** 2018-11-14

**Authors:** Akihiro Tamura, Suguru Uemura, Nobuyuki Yamamoto, Atsuro Saito, Aiko Kozaki, Kenji Kishimoto, Toshiaki Ishida, Daiichiro Hasegawa, Haruka Hiroki, Tsubasa Okano, Kohsuke Imai, Tomohiro Morio, Hirokazu Kanegane, Yoshiyuki Kosaka

**Affiliations:** 1 Department of Hematology and Oncology, Children’s Cancer Center, Kobe Children’s Hospital, Minatojima-Minamimachi 1-6-7, Chuo-ku, Kobe, 650-0047 Japan; 2 Department of Pediatrics, Graduate School of Medicine, Kobe University Hospital, Kusunoki-cho 7-5-2, Chuo-ku, Kobe, 650-0017 Japan; 3 Department of Pediatrics and Developmental Biology, Graduate School of Medical and Dental Sciences, Tokyo Medical and Dental University, Yushima 1-5-45, Bunkyo-ku, Tokyo, 113-8519 Japan; 4 Department of Community Pediatrics, Perinatal and Maternal Medicine, Graduate School of Medical and Dental Sciences, Tokyo Medical and Dental University, Yushima 1-5-45, Bunkyo-ku, Tokyo, 113-8519 Japan; 5 Department of Child Health and Development, Graduate School of Medical and Dental Sciences, Tokyo Medical and Dental University, Yushima 1-5-45, Bunkyo-ku, Tokyo, 113-8519 Japan

**Keywords:** Hematopoietic cell transplantation, Asymptomatic, X-linked lymphoproliferative disease type 1

## Abstract

**Background:**

X-linked lymphoproliferative disease type 1 (XLP1) is a rare primary immune deficiency, which is caused by *SH2D1A* gene mutations. XLP1 is commonly associated with Epstein–Barr virus (EBV)-associated hemophagocytic lymphohistiocytosis, hypogammaglobulinemia, and/or lymphoma. The only curative treatment for XLP1 is allogeneic hematopoietic cell transplantation. However, published data detailing the clinical course of, and indications for, allogeneic hematopoietic cell transplantation in asymptomatic patients with XLP1 is lacking. Although relevant family history could be useful in identifying patients with XLP1 before disease onset, no guidelines have been established on the management of asymptomatic patients with XLP1. Therefore, clinicians and families face dilemmas in balancing between the risk of waiting for the disease onset, and the risk of transplant-related mortality associated with allogeneic hematopoietic cell transplantation, which is often performed at a very young age. We first describe the detailed clinical course of an asymptomatic patient with XLP1 who successfully underwent allogeneic hematopoietic cell transplantation.

**Case presentation:**

A boy was born at 39 weeks of gestation, weighing 3016 g at birth. He appeared fine, but he underwent genetic testing because his maternal cousin had XLP1. He was found to have a novel c.207_208insC (p.Pro70ProfsX4) mutation in exon 3 of *SH2D1A*, which was also found in his cousin. There was no HLA-identical donor in his family. Immunoglobulin was administered monthly to prevent EBV infection while searching for an alternative donor. He underwent allogeneic bone marrow transplantation (BMT) from an allele HLA 8/8 fully matched, unrelated donor with a reduced-intensity conditioning (RIC) regimen consisting of fludarabine, melphalan, and low-dose total body irradiation (TBI) at 20 months of age. The patient has been doing well for 2 years post transplantation and maintaining complete donor chimerism without any evidence of chronic graft versus host disease.

**Conclusions:**

We describe a case of an asymptomatic patient with XLP1, who successfully underwent unrelated BMT with RIC regimen consisting of fludarabine, melphalan, and 3 Gy TBI. That was well tolerated and successfully generated complete chimerism in every subpopulation. This case delineates the option of allogeneic hematopoietic cell transplantation even in asymptomatic patients with XLP1.

## Background

X-linked lymphoproliferative disease type 1 (XLP1) is a rare primary immune deficiency that affects one in 1 million boys [1, 2]. XLP1 is caused by mutations in the *SH2D1A* gene that encodes the signaling lymphocytic activation molecule-associated protein (SAP) and is located on Xq25 [3]. SAP modulates intracellular signal transduction via its association with the SLAM family [4]. To date, more than 70 *SH2D1A* mutations have been reported with inconsistent genotype–phenotype correlation, which can vary even amongst family members who share the same mutation [2, 5]. XLP1 is commonly associated with Epstein–Barr virus (EBV)-associated hemophagocytic lymphohistiocytosis (HLH), hypogammaglobulinemia, and/or lymphoma [6, 7].

The only curative treatment for XLP1 is allogeneic hematopoietic cell transplantation (HCT). However, performing HCT after lymphoproliferation results in suboptimal outcomes [8]. Although relevant family history could be useful in identifying patients affected with XLP1 before disease onset, there are no guidelines on the management of asymptomatic patients with XLP1. Furthermore, no published data detail the clinical course of, and indications for, HCT for asymptomatic patients with XLP1. Therefore, clinicians and families face dilemmas in balancing between the risk of waiting for the disease onset, and the risk of treatment related mortality associated with allogeneic hematopoietic cell transplantation, which is often performed at a very young age. Furthermore, the optimal pretransplant conditioning regimen for XLP1 has not yet been established.

Here, we detail the clinical course of an asymptomatic patient with XLP1 with a novel c.207_208insC (p.Pro70ProfsX4) mutation in *SH2D1A* who successfully underwent allogeneic HCT with a reduced-intensity conditioning (RIC) regimen.

## Methods

*SH2D1A* mutation was detected by direct sequencing as previously described [9]. SAP protein expressions were assessed by flow cytometry as previously reported [10–13].

## Case presentation

**Fig. 1:**
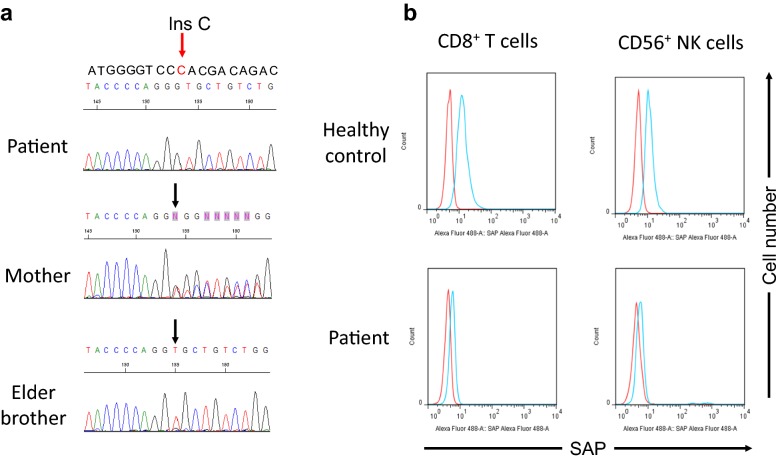
**a** Mutation analysis of XLP1 family. Direct sequencing analysis of the *SH2D1A* gene revealed c.207_208insC in the patient (upper panel). His mother showed a heterozygous mutation, indicating a carrier, whereas his elder brother showed a wild allele, indicating a normal (middle and lower panels). Arrows indicate the mutation site. **b** Flow cytometric analysis of SAP expression. Intracellular SAP expression in CD8^+^ T cells and CD56^+^ NK cells was reduced in the patient. Red line, isotype control; blue line, anti-SAP monoclonal antibody

A boy was born at 39 weeks of gestation, weighing 3016 g at birth. He appeared fine, but he underwent genetic testing because his maternal cousin had XLP1, and he was found to have a c.207_208insC (p.Pro70ProfsX4) mutation in exon 3 of *SH2D1A* (Fig. 1a, upper panel), which was the same mutation as that carried by his cousin. Gene analysis revealed that his mother was a carrier, and his elder brother was healthy (Fig. 1a, middle and lower panels). c.207_208insC in *SH2D1A* identified in this family was a novel mutation. SAP protein expressions assessed by flow cytometry were reduced both in CD8^+^ T cells and CD56^+^ NK cells (Fig. 1b). Serum immunoglobulin levels were of 275 mg/dL, 19 mg/dL, and 61 mg/dL for IgG, IgA, and IgM, respectively, at 6 months of age, which were within normal range for infants of this age [14]. There was no HLA-identical HCT donor in his family. Immunoglobulin was administered monthly to prevent EBV infection, although the utility of such prophylaxis has not been demonstrated. He was admitted to our hospital for allogeneic bone marrow transplantation (BMT) at 20 months of age. Blood cell and differential counts were within normal ranges with a white blood cell count of 9.6 × 10^9^/L (20.5% neutrophils, 1.5% eosinophils, 0.5% basophils, 4.0% monocytes, and 73.5% lymphocytes), a hemoglobin level of 9.7 g/dL, and a platelet count of 429 × 10^9^/L. Flow cytometric analysis of peripheral blood lymphocyte subsets revealed the following: CD3^+^ T cells: 57.0%; CD4^+^ T cells: 38.3%; CD8^+^ T cells: 20.8%; CD4/CD8 = 1.84; CD19^+^ B cells 9.2%; and CD56^+^ CD3^−^ NK cells 4.8%. NK cytotoxicity activity was assessed using Cr^51^ release assay, which was normal at 51.1%. The result of the phytohemagglutinin stimulation test was within the normal range. Our patient underwent allogeneic BMT from an allele HLA 8/8 fully matched, unrelated donor. The conditioning regimen consisted of the administration of 30 mg/m^2^ fludarabine for 5 days (days-8, -7, -6, -5, -4), 70 mg/m^2^ melphalan for 2 days (days-3, -2), and 3 Gy TBI (day-1). Graft versus host disease (GVHD) prophylaxis consisted of tacrolimus and short-term methotrexate. Engraftment of neutrophil was achieved on day 17. The patient developed acute GVHD grade I (skin stage 1), which spontaneously resolved over the clinical course. The patient had transient asymptomatic cytomegalovirus and aspergillus antigenemia. Otherwise, the BMT was uncomplicated. Chimerism analysis assessed by the recently developed mutation-specific droplet digital PCR showed more than 99% of donor type in every subpopulation (whole blood cells, peripheral blood mononuclear cells, granulocytes, T cells, and NK cells) as we recently reported [15]. He has been doing well for 2 years post transplantation and maintaining complete donor chimerism without any evidence of chronic GVHD.

## Discussion and conclusions

Here, we describe a case of an asymptomatic patient with XLP1 with c.207_208insC mutation, who successfully underwent unrelated BMT with RIC regimen consisting of fludarabine, melphalan, and 3 Gy TBI. A recent international retrospective study showed an excellent survival rate (93%; 15 out of 16) in asymptomatic patients with primary HLH (*PRF1*, *STXBP2*, *UNC13D*, *STX11*, *RAB27A*, and *LYST* deficiency) treated with HCT [16]. Asymptomatic patients with XLP1 would have better HCT outcome as well as those with primary HLH. In the largest retrospective analysis of transplantation for XLP1, all patients who underwent allogeneic HCT before the onset of HLH survived (27 out of 27), whereas patients who underwent HCT after HLH onset resulted in decreased survival rate of 50% [8]. Even in the largest retrospective study, indications for HCT, besides HLH, were lymphoma and/or dysgammaglobulinemia [8]. It is not clear whether asymptomatic patients underwent HCT as part of this study [8]. Two asymptomatic patients with XLP1 underwent HCT in Japan previously; however, the detailed clinical information is lacking for the two other Japanese asymptomatic patients with XLP1 [5] (Table 1). Despite variable conditioning regimens, all three patients including our patient survived without chronic GVHD (Table 1). The prognosis of patients with XLP1 not undergoing HCT is poor [8]. In contrast, the prognosis is good in patients with XLP1 who undergo HCT before disease onset. Therefore, we suggest that HCT should be performed even in asymptomatic patients.

**Table 1:** HCT for asymptomatic patients with XLP1

Age	Donor Source	HLA matching	Conditioning regimen	GVHD prophylaxis	aGVHD	cGVHD	Outcome	References
1 years	Unrelated BM	6/6	Bu/TAI 3 Gy/Flu/CY/ATG	Tacrolimus/sMTX	None	None	Alive (3 years)	[5]
8 months	Unrelated PBSC	5/6	Flu/Mel/ATG/TAI 6 Gy	Tacrolimus/sMTX/PSL	GradeII	None	Alive (9 years)	[5]
20 months	Unrelated BM	8/8	Flu/Mel/TBI 3 Gy	Tacrolimus/sMTX	GradeI	None	Alive (2 years)	Our patient

*aGVHD* acute graft versus host disease, *ATG* anti thymocyte globulin, *BM* bone marrow, *Bu* busulfan, *cGVHD* chronic graft versus host disease, *CY* cyclophosphamide, *Flu* fludarabine, *Mel* melphalan, *PBSC* peripheral blood stem cells, *PSL* prednisolone, *sMTX* short term methotrexate, *TAI* total abdominal irradiation, *TBI* total body irradiation

Furthermore, the optimal pretransplant conditioning regimen for XLP1 has not yet been established because the number of patients with XLP1 is limited. Historically, most patients with XLP1 have been transplanted using myeloablative-conditioning regimens [17]. Recently, a RIC regimen has been used to reduce therapy-related mortality and late sequelae [5, 17]. Marsh et al. [18] reported promising results with a 75% (12 of 16) survival rate in the treatment of patients with XLP1 with a RIC regimen consisting of alemtuzumab, fludarabine, and melphalan. However, in this study, a high rate of mixed chimerism (5 of 16 patients) and infections (3 of 4 deaths attributed to infectious complications) are noted [18]. In our patient, complete chimerism (> 99%) for every subpopulation was confirmed using the recently developed mutation-specific droplet digital PCR [15]. In Japan, 11 of 12 patients (92%) with XLP1 who underwent HCT with various conditioning regimens survived [5]. In this previous report, three patients underwent transplantation with RIC regimen consisting of fludarabine, melphalan, and low-dose TBI, and all patients are alive (Table 2). Alemtuzumab is not approved in the setting of pretransplant conditioning in Japan and in many other countries; therefore, fludarabine, melphalan, and low-dose TBI can be a good candidate for conditioning regimen for XLP1. Further clinical studies are required to establish optimal strategy of transplantation for patients with XLP1.

**Table 2:** Flu/Mel/TBI 3 Gy conditioning HCT for XLP1 in Japan

Age	Symptoms	Donor Source	HLA matching	GVHD prophylaxis	aGVHD	cGVHD	Outcome	References
3 years	HLH, hypo-γ	Unrelated BM	4/6	Tacrolimus/sMTX	Grade I	None	Alive (8 years)	[5]
7 years	Hypo-γ	Unrelated BM	6/6	Tacrolimus/sMTX	None	Extensive	Alive (4 years)	[5]
15 years	HLH, ML, hypo-γ	Unrelated BM	6/6	Tacrolimus/sMTX	None	None	Alive (3 years)	[5]
20 months	Asymptomatic	Unrelated BM	8/8	Tacrolimus/sMTX	Grade I	None	Alive (2 years)	Our patient

*HLH* hemophagocytic lymphohistiocytosis, *hypo-γ* hypo gammaglobulinemia

## Abbreviations

XLP1: X-linked lymphoproliferative disease type 1
SAP: signaling lymphocytic activation molecule-associated protein
EBV: Epstein–Barr virus
HLH: hemophagocytic lymphohistiocytosis
HCT: hematopoietic cell transplantation
RIC: reduced-intensity conditioning
TBI: total body irradiation
BMT: bone marrow transplantation
GVHD: graft versus host disease
